# An atypical case of infection with the Mpox virus clade IIb lineage C.1 – clinical and virological findings

**DOI:** 10.1007/s10096-025-05346-8

**Published:** 2025-11-18

**Authors:** Pawel Zmora, Blazej Rozplochowski, Monika Gazecka, Szymon Nowak, Dagny Lorent, Arleta Kowala-Piaskowska

**Affiliations:** 1 Department of Molecular Virology, Institute of Bioorganic Chemistry Polish Academy of Sciences, Poznan, Poland; 2 Department of Infectious Diseases, Hepatology and Acquired Immunodeficiencies, Poznan University of Medical Sciences, Poznan, Poland; 3 Department of Internal Medicine, Poznan University of Medical Sciences, Poznan, Poland

**Keywords:** Mpox, Mpox virus (MPXV), Clade IIb, Lineage C.1, Phylogenomic, Atypical course of infection

## Abstract

We describe an atypical case of mpox virus (MPXV) infection in a Polish patient, with a focus on clinical presentation, virological characterisation, and the phylogenetic placement of the viral isolate within the emerging C.1 lineage of Clade IIb. The report aims to contribute to a better understanding of MPXV lineage-specific disease features and viral evolution in non-endemic settings. The patient presented with numerous localised lesions and minimal systemic symptoms. Genomic analysis confirmed infection with an MPXV C.1 lineage of Clade IIb responsible for the 2022–2025 global outbreaks. Infection with C.1 lineage may lead to atypical presentations, particularly in immunocompetent individuals. Continued genomic surveillance and clade-specific clinical correlation are essential for accurate diagnosis, risk stratification, and public health response.

## Introduction

Mpox (*formerly monkeypox*) virus (MPXV), an orthopoxvirus closely related to variola virus, has been transformed from a virus endemic to Central and Western Africa, with some skin lesions as infection manifestation, to a critical global health concern, following its 2022–2025 emergence in non-endemic countries. Between January 2022 and March 2025, over 137,000 confirmed cases across 132 countries and 317 deaths were reported worldwide [1]. In Europe and the EU/EEA, the cumulative number of confirmed cases reached approximately 24,772 as of June 19, 2025, with 245 reported in Poland alone [1]. However, as we showed, these numbers may be highly underestimated due to the social stigmatisation, lack of trust in public healthcare systems, as well as very mild or even asymptomatic outcomes of MPXV infection [2]. This means that, although classified as a Public Health Emergency of International Concern in both July 2022 and August 2024 [1], the mpox outbreak seems not to have ended shortly.

The MPOX outcome may differ, since MPXV is genetically divided into two primary clades, i.e., Clade I and Clade II, with distinct epidemiological and clinical profiles [1, 3, 4]. Clade I (formerly Central African/Congo Basin clade) is associated with higher virulence, case fatality rates up to 10%, more frequent human-to-human transmission, and systemic illness with disseminated lesions [4]. Recently, a Clade Ib has emerged and caused a major epidemic in Central Africa since September 2023, with over 29 thousand suspected cases, ~ 800 deaths and a case fatality rate (CFR) ~ 3% [1]. Moreover, MPXV Clade Ib infections showed distinct clinical patterns, i.e., genitourinary involvement in adults, compatible with greater sexual transmission, and more widespread lesions manifested mostly extragenitally in children [4]. Finally, Clade Ib started to spread globally, with mostly isolated, imported, and travel-linked cases confirmed in Sweden, Thailand, the U.S.A., Canada, Germany, China, and France. The first local MPXV Clade Ib transmissions were described recently in the UK [1]. On the other hand, Clade II (formerly West African clade), which includes Clade IIb responsible for the 2022–2025 global outbreaks, shows lower virulence, CFR < 1% and often manifests with single localised lesions, mainly anogenital, sometimes without prodromal symptoms. It is thought that Clade IIb is more adapted to human-to-human sexual transmission, in particular in MSM networks [4].

Despite these multiregional findings, reports of atypical clinical presentations have increased, complicating diagnostic and containment efforts. Manifestations now often include isolated mucosal lesions, delayed rash development, or absence of systemic prodromes, features that can mimic other conditions and lead to misdiagnoses [1, 3].

Here, we describe a particularly atypical MPXV infection in a Polish patient, emphasising both clinical and virological findings that diverge from established patterns. This case not only highlights the evolving epidemiological profile of mpox in non-endemic regions but also underscores the imperative for vigilant clinical suspicion, comprehensive diagnostic protocols, and integrated STI screening and management in affected populations.

## Case presentation

A 25-year-old cisgender man who has sex with men (MSM), presented to the Department of Infectious Diseases, Hepatology and Acquired Immunodeficiencies, Poznan University of Medical Sciences (DIDHAI PUMS), on February 8, 2025, with a 6-day history of systemic and mucocutaneous symptoms. He reported an acute febrile illness beginning between February 2 and 4, with temperatures reaching 39 °C and accompanied by a sore throat. Initially, the patient was evaluated by a primary care physician and received empirical treatment with cefuroxime (500 mg bid, administered orally) for suspected pharyngitis, without microbiological testing. Fever resolved by February 5, but he noted the onset of skin lesions on the same day.

Patient denied any travel in the months preceding symptom onset but admitted unprotected sexual contact with new male partners approximately two weeks before the disease’s symptoms manifested.

Patient’s medical history was notable for an HIV diagnosis in October 2024. Antiretroviral therapy with bictegravir/emtricitabine/tenofovir alafenamide was initiated shortly after diagnosis. Subsequent viral load monitoring showed suppression to undetectable levels by November 2024 and remained suppressed through March 2025. On June 17, 2025, a HIV viremia of 25.8 copies/mL was detected, with a CD4 count of 568/µL and a CD4/CD8 ratio of 0.58.

**Fig. 1:**
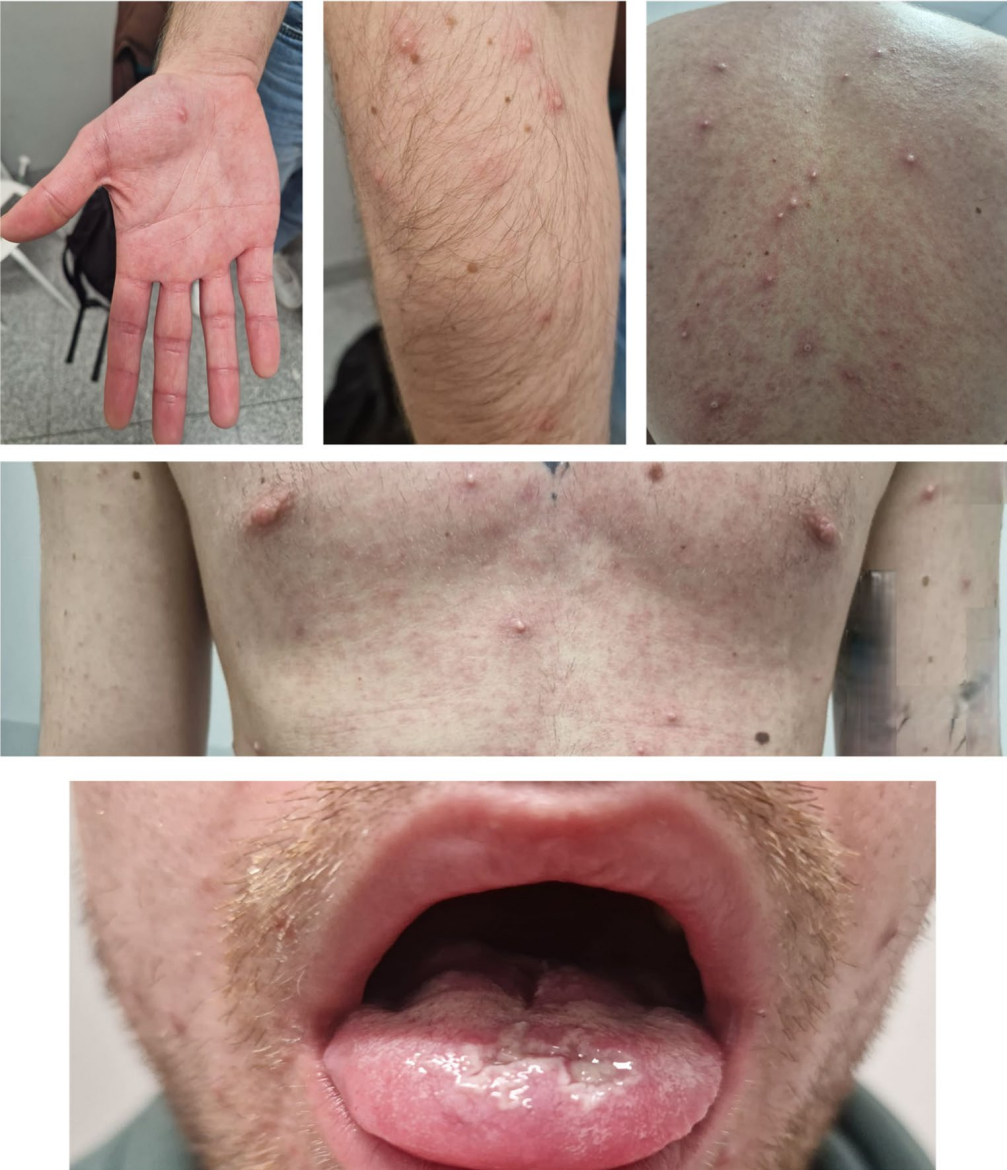
Widespread vesiculopustular lesions with serous to turbid content, affecting the entire body, including the right hand (upper left panel), right forearm (upper middle panel), back (upper right panel) and chest (middle panel). Mucosal involvement included erythematous and oedematous oropharynx, hypertrophic tonsils, and numerous erosions on the buccal mucosa and tongue (lower panel)

On the day of admission to the hospital (February 8), clinical examination revealed widespread vesiculopustular lesions with serous to turbid content, affecting the entire body, including the scalp, palms, soles, and genital area (Fig. 1). The lesions were particularly numerous on the buttocks. In addition, the patient exhibited a maculopapular erythematous rash on the trunk. Mucosal involvement included erythematous and edematous oropharynx, hypertrophic tonsils, and numerous erosions on the buccal mucosa and tongue. Mild cervical lymphadenopathy and slight bronchial murmurs were noted.

New vesicles continued to appear on February 11 and 12 (notably on the right elbow and knee), while previously formed lesions began to desiccate. By February 14, all lesions had crusted, and the patient was discharged in good general condition.

The patient was treated empirically with doxycycline and cefazolin, in addition to topical antifungal (nystatin) and symptomatic therapy. Clinical improvement was noted during hospitalisation, with complete lesion resolution by discharge.

**Fig. 2:**
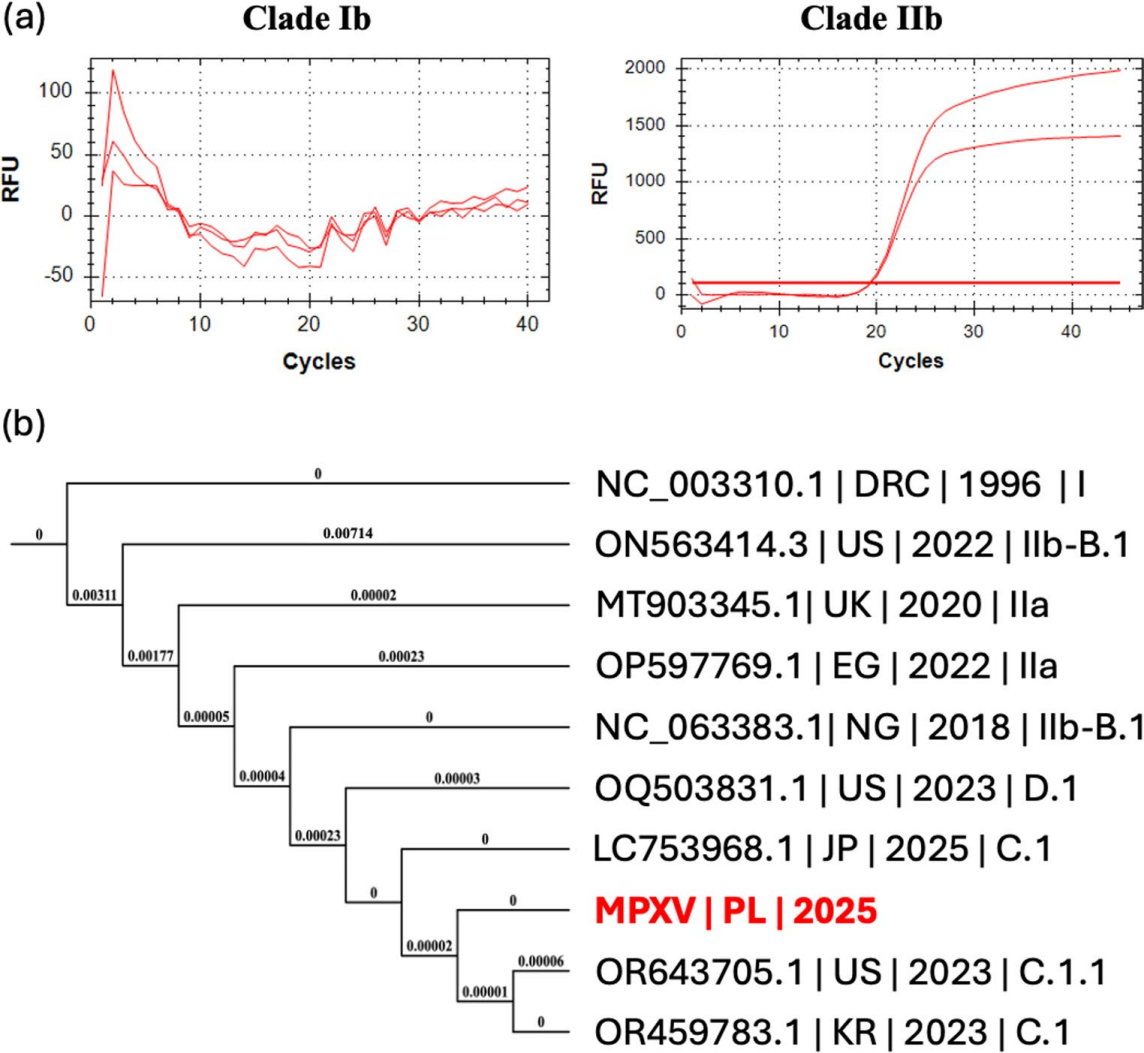
Identification of MPXV clade and lineage by (**a**) PCR and (**b**) Next Generation Sequencing (NGS). (**a**) Amplification curves for MPXV clade Ib (left panel) and MPXV clade IIb (right panel) for the PCR with MPXV clade-specific primers and probes. (**b**) The Maximum-Likelihood Tree for the genome sequences of MPXV isolated from the described patient (marked with red) and some reference isolates. DRC – Democratic Republic of Congo (formerly: Zaire), US – United States of America, UK – United Kingdom of Great Britain and Northern Ireland, EG – Egypt, NG – Nigeria, JP – Japan, PL – Poland, KR – Republic of Korea

Taken together, the distribution and progression of mucocutaneous lesions, history of MSM exposure, and concurrent HIV infection, these findings were highly suggestive of MPXV infection. Moreover, due to the atypical manifestation of symptoms, as well as the first cases of MPXV Clade Ib infections in neighbouring countries, such as Germany and the Czech Republic, we suspected the first case of mpox caused by MPXV Clade Ib in Poland. To confirm the emergence of MPXV Clade Ib in Poland, skin swabs were taken from the patient, inactivated and transferred from the DIDHAI PUMS, to the Department of Molecular Virology, Institute of Bioorganic Chemistry Polish Academy of Sciences. Subsequently, we isolated DNA from the samples using the NucleoSpin Blood, Mini kit for DNA from blood (Macherey Nagel, Germany), and conducted real-time PCR with MPXV Clade Ib-specific [5] and MPXV Clade IIb-specific (ThermoFisher, Germany) primers and probes. Amplification of the MPXV genetic material occurred exclusively for Clade IIb (Fig. 2a). Our results were subsequently confirmed by the reference laboratory at the National Institute of Public Health – National Institute of Hygiene.

To further understand the cause of the atypical MPXV infection course, we used Next Generation Sequencing, i.e., Oxford Nanopore Technologies, to sequence the MPXV whole genome. A complete genome of MPXV, containing 195,846 base pairs, was provided in FASTA format and deposited in GenBank with accession PX067946.1, The obtained sequence was screened for known lineage-defining single-nucleotide polymorphisms (SNPs) associated with major lineages of Clade IIb, particularly within the B.1, C.1, C.1.1, B.1.23, D.1, E.1-E.3 and F.1-F.6 lineages. As a result, the query genome matched all nine defining SNPs for lineage C.1, corresponding to a 100% identity score. Finally, a simplified multiple sequence alignment was generated using reference sequences for Clade I, Clade IIa, Clade IIb lineages B.1, C.1, C.1.1 and D.1. A phylogenetic tree was constructed using the Maximum Likelihood method and exported in Newick format. The tree topology was used to confirm lineage placement of the query genome. In the maximum likelihood tree, the query sequence clustered tightly with reference genomes of C.1, forming a monophyletic branch distinct from C.1 and C.1.1 and supporting the SNP-based assignment (Fig. 2B). Finally, comparative genomic analysis of the C.1 isolate revealed several non-synonymous mutations affecting genes involved in host immune evasion and virulence modulation, including: *B21R* (surface glycoprotein, immune evasion), *OPG210* (TNF receptor homolog), *A46R* (inhibitor of TLR signalling), and *CrmB* and *CrmC* (complement-binding proteins). These mutations may attenuate or modulate host immune responses, potentially contributing to the altered clinical presentations observed in Clade IIb infections.

## Discussion

This case of atypical MPXV infection in a Polish patient highlights the increasingly diverse clinical manifestations of mpox, especially in non-endemic regions. Several key findings emerge from our analysis, including host-related risk factors, clade-specific virological characteristics, and notable mutational signatures in the viral genome that may have implications for pathogenicity and transmission.

The clinical outcome of mpox varies considerably depending on host factors, particularly immunological status. People living with HIV, especially those with low CD4 counts or unsuppressed viral loads, have consistently been shown to be at higher risk for severe, prolonged, or disseminated MPXV infection. Similarly, patients undergoing chemotherapy or those with other forms of immunosuppression, such as organ transplant recipients or individuals with haematological malignancies, may present with atypical or fulminant disease [3]. In our case, the patient was immunocompetent, which may partially explain the self-limited course despite the isolate belonging to a lineage Clade C.1 with limited published data regarding clinical severity. The interplay between host immunity and viral genetics is crucial and warrants continued investigation.

Furthermore, the virus isolated from this patient was assigned to the C.1 lineage of Clade IIb, based on whole genome sequencing and phylogenetic analysis. The C.1 lineage emerged during the 2022–2023 global outbreak, primarily in Europe, and represents one of the most genetically divergent clusters within Clade IIb. This lineage is characterised by a set of accumulated SNPs and APOBEC3-driven mutations, which suggest ongoing viral adaptation to human-to-human transmission. The implications of a C.1 classification are multifaceted: (1) while Clade IIb (including C.1) is associated with milder disease compared to Clade I, the symptom spectrum appears broader, with a higher frequency of localised lesions, isolated mucosal involvement, and asymptomatic or paucisymptomatic cases; (2) although current evidence does not definitively link C.1 to increased severity, its efficient transmission in MSM networks, particularly through close contact or sexual activity, raises public health concerns.

Interestingly, when compared to the recently emerged Clade Ib, our C.1 isolate shares some molecular and phenotypic similarities, e.g., both show evidence of APOBEC3-mediated mutational pressure, indicating adaptive evolution within human hosts [4]. In addition, both clades include strains with enhanced transmissibility in adult sexual networks, and reports of atypical presentations including isolated genital lesions and mucosal tropism [6]. However, Clade Ib has been associated with higher case fatality rates (~ 3%), broader tissue tropism, and mixed presentation patterns in both adults and children, especially in endemic settings such as the Democratic Republic of Congo [7, 8]. Clade C.1 has not, to date, demonstrated this level of virulence, although continued surveillance is needed.

In conclusion, our findings reinforce the importance of integrating clinical, epidemiological, and genomic data to understand the evolving dynamics of MPXV infection. Lineage C.1, while not associated with severe disease in this case, exhibits signs of ongoing adaptation, warranting close monitoring. Future studies should focus on the functional consequences of specific mutations, host-virus interactions in immunocompromised populations, and the comparative virulence of emerging lineages such as Clade Ib and C.1 in both endemic and non-endemic regions. We are also aware that this report is limited by its single-patient nature; causality between viral mutations and clinical phenotype cannot be inferred, especially if the deposited MPXV genome in the NCBI Virus – MPXV Data Hub is the first and so far only MPXV sequence from Poland. However, as more Clade C.1 genomes are characterised, correlational studies between clades/lineages and clinical severity or tropism may become feasible and should be prioritised.

## Data Availability

Genomic sequence of MPXV|Poland|Poznan|2025|PZ01 was deposited into the NCBI GenBank under accession number PX067946.1.
